# Supplementary figures and images for: Molecular Analysis of SARS-CoV-2 Spike Protein-Induced Endothelial Cell Permeability and vWF Secretion
Source: Int J Mol Sci. 2023 Mar 16;24(6):5664. doi: 10.3390/ijms24065664 (PMC10053386; doi:10.3390/ijms24065664)

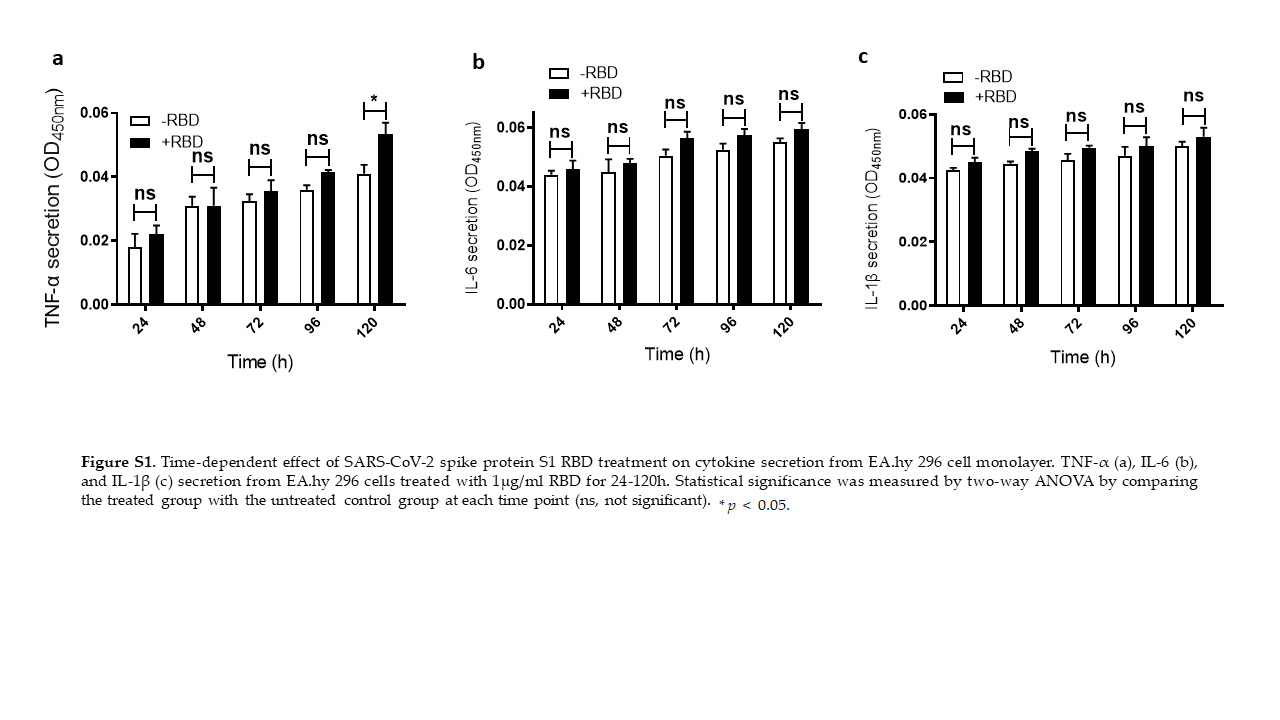

Supplement: Supplementary file 1 [file ijms-24-05664-s001.zip › Figure S1.TIF]

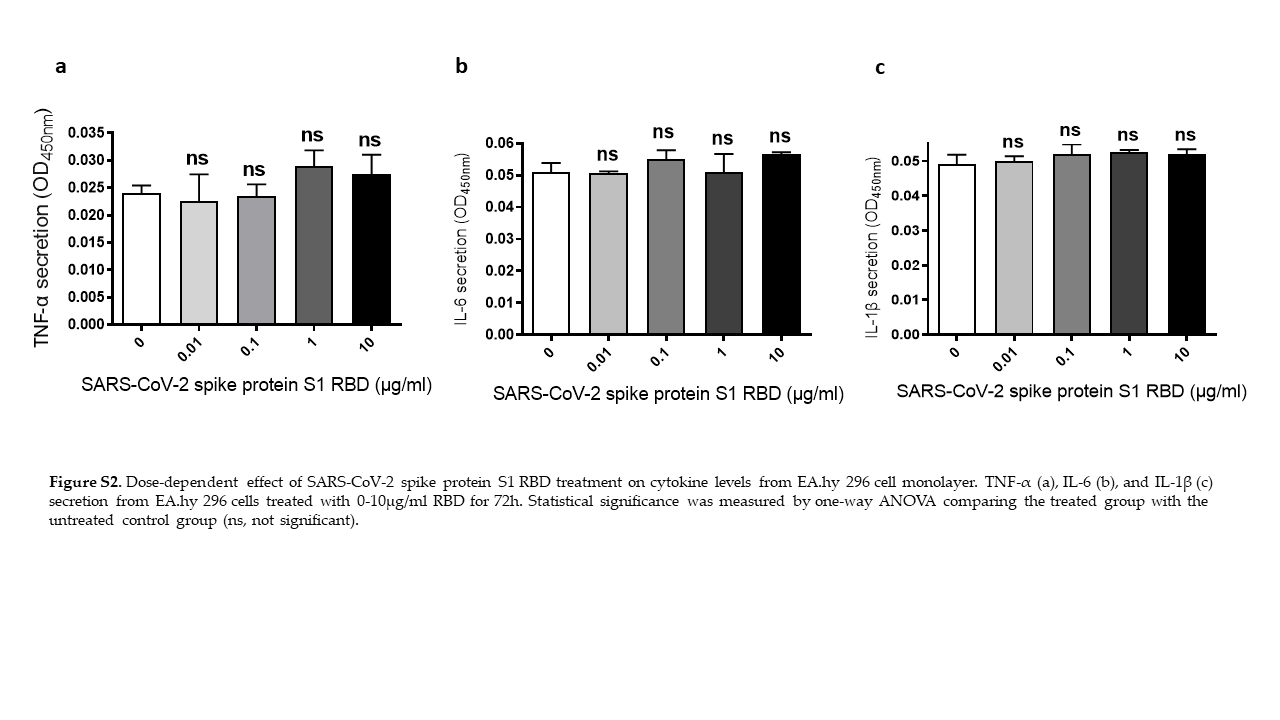

Supplement: Supplementary file 1 [file ijms-24-05664-s001.zip › Figure S2.TIF]

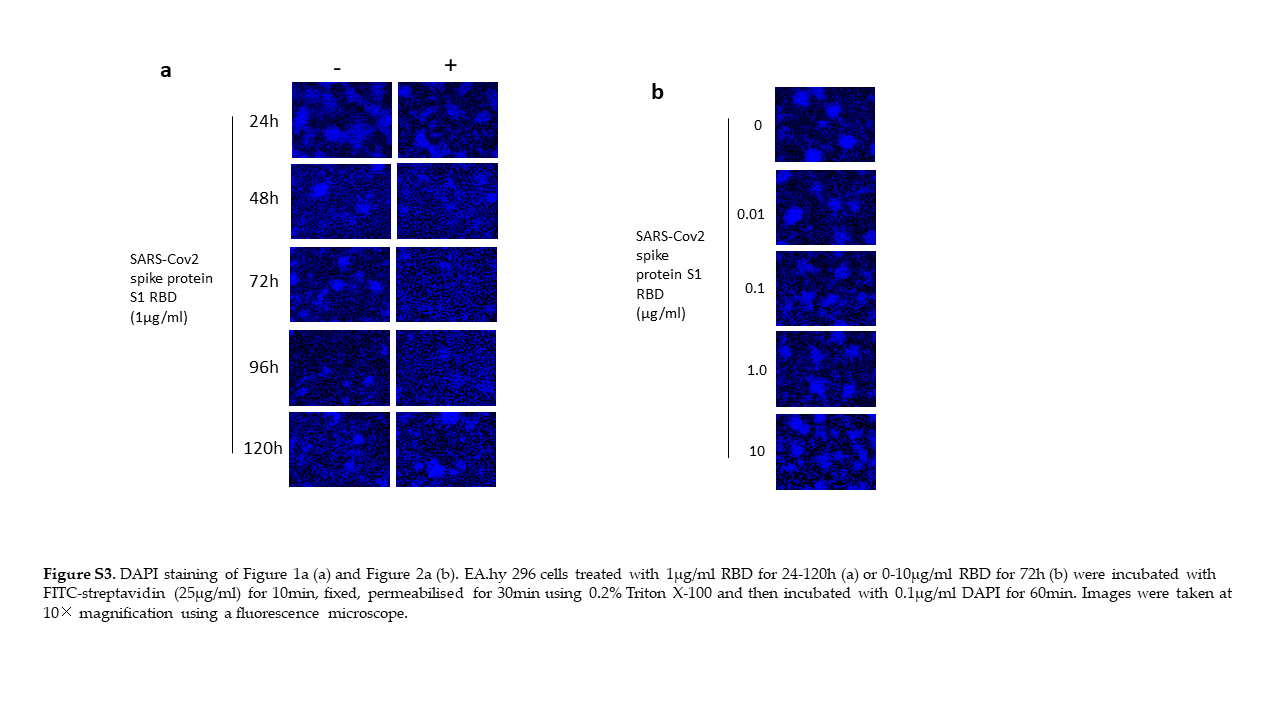

Supplement: Supplementary file 1 [file ijms-24-05664-s001.zip › Figure S3.tif]
